# Socioeconomic vulnerability and differential impact of severe weather-induced power outages

**DOI:** 10.1093/pnasnexus/pgad295

**Published:** 2023-10-03

**Authors:** Scott C Ganz, Chenghao Duan, Chuanyi Ji

**Affiliations:** McDonough School of Business, Georgetown University, 37th and O Streets, NW, Washington, DC 20057, USA; American Enterprise Institute, 1789 Massachusetts Ave NW, Washington, DC 20036, USA; School of Electrical and Computer Engineering, Georgia Institute Technology, Atlanta, GA 30332, USA; School of Electrical and Computer Engineering, Georgia Institute Technology, Atlanta, GA 30332, USA

**Keywords:** energy equity, natural disasters, power outages, spatial data analysis

## Abstract

In response to concerns about increasingly intense Atlantic hurricanes, new federal climate and environmental justice policies aim to mitigate the unequal impact of environmental disasters on economically and socially vulnerable communities. Recent research emphasizes that standard procedures for restoring power following extreme weather could be one significant contributor to these divergent outcomes. Our paper evaluates the hypothesis that more economically and socially vulnerable communities experience longer-duration power outages following hurricanes than less vulnerable communities do, conditional on the severity of the impact of the storm itself. Using data from eight major Atlantic hurricanes that made landfall between January 2017 and October 2020 and induced power outages for over 15 million customers in 588 counties in the Southeast, we demonstrate a significant relationship between socioeconomic vulnerability and the duration of time that elapses before power is restored for 95% of customers in a county. Specifically, a one-decile change in the socioeconomic status theme in the Social Vulnerability Index, a measure of vulnerability produced by the Centers for Disease Control and Prevention and the Agency for Toxic Substances and Disease Registry, produces a 6.1% change in expected outage duration in a focal county. This is equivalent to a 170-min average change in the period of time prior to power restoration.

**Significance Statement**

Using data from eight major Atlantic hurricanes that made landfall between January 2017 and October 2020, we demonstrate a significant relationship between socioeconomic vulnerability and the duration of time that elapses before power is restored for 95% of customers in a county. A one-decile change in the socioeconomic status theme in the Social Vulnerability Index produces a 6.1% change in expected outage duration in a focal county, which is equivalent to a 170-min average change in the period of time prior to power restoration.

## Introduction

In response to concerns about increasingly intense Atlantic hurricanes, new federal climate and environmental justice policies aim to mitigate the impact of environmental disasters on economically and socially vulnerable communities (1, 2). The necessity of these policy initiatives is supported by a series of recent statistical case studies that demonstrate that more vulnerable communities experience more hardship as a result of hurricanes (3–7). Success in achieving these policy goals depends on identifying the features of storms, communities, and policies that contribute most to this unequal impact and designing targeted interventions to improve outcomes for vulnerable communities (8–11).

Recent research emphasizes that standard procedures for restoring power following extreme weather could be one significant contributor to these divergent outcomes. These procedures, which first prioritize critical infrastructure, then commercial and industrial firms, and then seek to recover as many households as quickly as possible (12, 13), are indifferent to the economic and social vulnerability of communities and households (14). Because of long-standing inequity in investment in critical infrastructure and other public services and also because more economically and socially vulnerable communities tend to be more physically distant from commercial and industrial centers, these policies have the potential to produce longer duration outages in disadvantaged communities than in better-resourced ones (15–17). Although *procedurally fair*, the recovery routines in widespread use throughout the United States could exacerbate existing sources of inequality.

Our paper evaluates the hypothesis that more economically and socially vulnerable communities experience longer duration power outages following hurricanes than less vulnerable communities do, conditional on the severity of the impact of the storm itself. Using data from eight major Atlantic hurricanes that made landfall between January 2017 and October 2020 and caused power outages for over 15 million customers in 588 counties in the Southeast, we demonstrate a significant relationship between socioeconomic vulnerability and the duration of time that elapses before power is restored for 95% of customers in a county. Specifically, a one-decile change in the socioeconomic status theme in the Social Vulnerability Index (SVI), a measure of vulnerability produced by the Centers for Disease Control and Prevention and the Agency for Toxic Substances and Disease Registry (CDC/ATSDR), produces a 6.1% change in expected outage duration in a focal county. This is equivalent to a 170-min average change in the period of time prior to power restoration. SVI themes related to household composition, racial & ethnic minority status, and housing type & transportation, in contrast, do not exhibit a statistically significant relationship with outage duration.

## Background

Our paper builds on decades of research in engineering and the social sciences that analyze the distributional effects of natural disasters on economically and socially vulnerable communities. Prior research demonstrates that vulnerable communities are less able to anticipate natural disasters, cope with their acute impact, and then recover from the damage they cause (18–21). This research details the characteristics of communities that lead them to experience greater hardship as a result of natural disasters, including poverty levels, percentages of racial or ethnic minorities, and concentrations of households with disabilities (10, 22). Recent studies show, for example, that people who report having poor health are less prepared for disasters (23), households in communities with smaller racial or ethnic minority populations evacuate farther from impacted areas prior to extreme weather events (24), and evacuated households in socioeconomically vulnerable communities are slower to return to their homes following a disaster (25).

Recent federal policies related to climate and environmental justice focus, in particular, on the intersection between economic and social vulnerability and the impact of climate change. The CDC/ATSDR SVI, which we use in our paper to measure the economic and social vulnerability of communities to prolonged storm-caused power outages, combines socioeconomic indicators related to poverty, housing costs, education, and health insurance, with additional indicators associated with household characteristics, racial & ethnic minority status, and housing type & transportation in order to “assess community need during emergency preparedness planning” and “identify communities that will need continued support to recover following an emergency or natural disaster” (26). The Council on Environmental Quality’s Climate and Economic Justice Screening Tool similarly determines community vulnerability based on direct storm-related risks (e.g. costs associated with of flooding and fires) together with socioeconomic indicators in order to “help identify disadvantaged communities that will benefit from programs included in the Justice40 Initiative,” which “seeks to deliver 40% of the overall benefits of investments in climate, clean energy, and related areas to disadvantaged communities” (27).

One of the most disruptive acute effects of hurricanes for economically and socially vulnerable households is long-duration power outages. Long-duration outages can impact health through, e.g. increased exposure to carbon monoxide, decreased access to clean water, consumption of spoiled food, or lack of shelter (28). Outages also cause significant disruptions in the provision of medical services as the number of individuals requiring care increases but the ability to provide electricity-dependent care is hindered (29). Proper preparation for long-duration outages can help mitigate these outcomes. But such preparation is less common for vulnerable communities, e.g. those with lower median incomes, higher median ages, and fewer native English speakers (30).

There is strong statistical evidence that more economically and socially vulnerable communities are more likely to experience extended power outages (see, e.g. (31, 32)). But, appropriate policy interventions depend on the extent to which these outages are the result of these communities being more likely to be located in a storm’s path or, instead, are the result of prolonged recovery processes conditional on the storm’s impact. Quantitative evidence on the relationship between economic and social vulnerability and power outage duration conditional on storm impact is mixed, with some demographic factors correlated with vulnerability predicting longer duration outages (and only after removing other insignificant covariates from the statistical model) (3) or after aggregating the direct effect of the vulnerability on a focal county with estimated spillover effects from the focal county to its neighbors (7).

However, existing case-based empirical analyses that focus on one storm in a limited geographic region face the difficult statistical challenge of disentangling the impact of the storm itself from the characteristics of communities impacted by the storm, two variables that exhibit strong spatial dependence (33, 34). What is required in order to identify the theorized relationship between economic and social vulnerability and power outage duration given the various sources of spatial correlation is a large-scale analysis that encompasses multiple comparable severe weather events impacting large geographic regions with communities characterized by widespread variation in economic and social vulnerability. These necessary features of the data make the Southeast United States an especially attractive site for study.

## Data

Our analysis combines large-scale data on storm-caused power outages in the Southeast with data on the economic and social vulnerability of affected communities, characteristics of hurricanes, and storm-related risks.

Our data on power failures span eight major Atlantic hurricanes that made landfall between 2017 and 2020 (35, 36). The hurricanes are selected from the recorded Atlantic hurricanes based on two criteria. First, the Saffir-Simpson Hurricane Wind Scales on the mainland of the Southeast were at least category 1. Second, the nominal costs ranked in the top 10 (with inflation-adjusted costs over $5 billions) (37) during the period from 2017 to 2020. We obtain data on power outages from PowerOutage.com (35), which compiles county-level, time-varying data on the number of customers affected by large-scale power outages in a region as reported by utilities.

We evaluate the impact of the hurricanes on nine states in the Southeast (Alabama, Arkansas, Florida, Georgia, Louisiana, Mississippi, North Carolina, South Carolina, and Tennessee). The dependent variable in our study is the duration of time that a county experiences a power outage following hurricane landfall. Outage duration for each affected county is computed as the duration of time that elapses between the time at which the number of disrupted customers reaches its peak level and the time it drops below 5% of the customers served. A county is thus included in our dataset if at least 5% of all customers served experienced power outages during a given hurricane. In our statistical analyses, we focus on outages that last for more than one hour, consistent with minimum duration for an outstanding outage that affected at least 50,000 customers to be considered an “emergency situation” by the US Department of Energy (38), although we also present results that reduce this minimum-duration threshold. Overall, the affected service regions span over 338 thousand square miles, covering 588 counties, 26.87 million customers served, and 167 affected utilities. Details on each hurricane are presented in Tables 1 and 2.

**Table 1.** Event data failure summary.

Event	Peak outages	Affected utilities	Wind
	(thousands)		(knots)
Florence	954.91	45	64
Harvey	17.83	10	34
Irma	7290.07	62	64
Isaias	364.51	31	50
Laura	637.19	22	64
Michael	734.57	56	64
Sally	548.82	34	64
Zeta	2158.36	70	64

*Note*: Peak outages: maximum number of customers in outage.

**Table 2.** Event data geographic summary.

Event	Category	Nominal cost (billions USD)	Affected southeastern states	Affected counties	Customers served (millions)
Florence	4	24.2	NC, SC	102	3.91
Harvey	4${}^{a}$	125${}^{a}$	LA, TN	48	0.67
Irma	5	31	AL, FL, GA, SC	315	15.04
Isaias	1	4.8	NC	62	1.55
Laura	4	19.1	AR, LA	116	2.76
Michael	5	25.5	AL, FL, GA	184	3.26
Sally	2	7.3	AL, FL	74	2.99
Zeta	3	4.4	AL, GA, LA, MS	237	8.62

${}^{a}$ Harvey incurred significant impact in Texas, which is not included in the analysis.

**Fig. 1.**
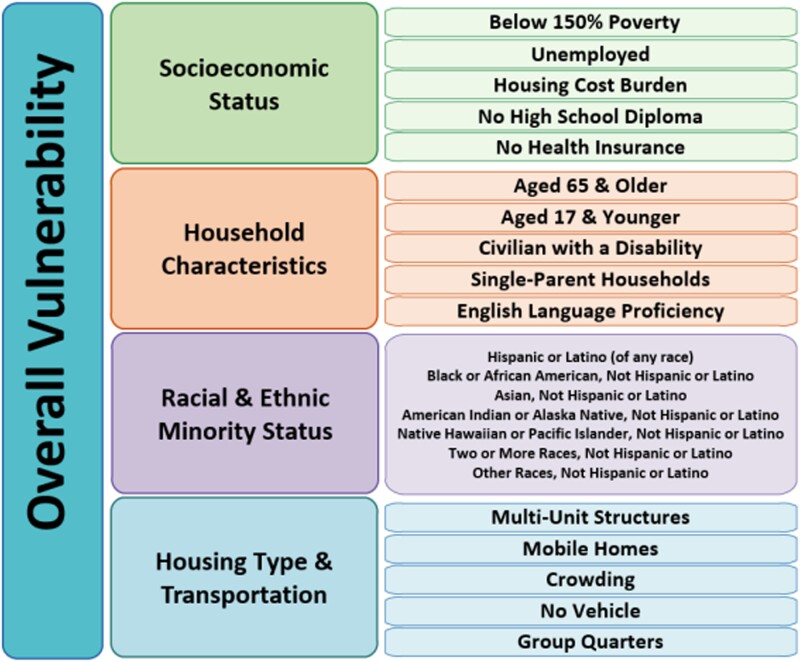
Themes and variables from American Community Survey used to compute the SVI for each county. *Source:* CDC SVI Documentation 2020 (26).

We measure the economic and social vulnerability of communities using the CDC/ATSDR SVI, computed on the county level (39). SVI, which is widely used in recent studies of the differential impact of strong storms [e.g. (3, 5, 7, 14, 40)], reports the percentile ranking of counties according to four vulnerability themes: Socioeconomic Status, Household Characteristics, Racial & Ethnic Minority Status, and Housing Type & Transportation (25). In order to indicate that higher values for each theme represent more vulnerability, we refer to the first theme as *Socioeconomic vulnerability*, the second as *Household characteristics vulnerability*, the third as *Racial & ethnic minority status vulnerability*, the fourth as *Housing type & transportation vulnerability*. We refer to the aggregate SVI index as *Overall social vulnerability*. Vulnerability for each theme is compiled using data from the American Community Survey on income level, age, language spoken at home, and type of housing, among others (see Fig. 1 for a complete list of variables for each theme). Each county *i* is ranked according to each of these 16 variables. A percentile rank of variable *v* is then computed for *i* as follows: percentile_rank_*i,v*_ = (rank_*i,v*_ − 1/(*N* − 1), where rank_*i,v*_ is the rank of *i* according to *v* and *N* is the total number of counties. For each of the four themes, the percentile rankings for each variable are summed to compute a theme-level index. To compute the overall SVI, the four theme-level indices are summed for each county, then each county receives a percentile ranking according to this sum. Note that, although the SVI can be computed on the level of the census tract, our ability to use more granular data on economic and social vulnerability is constrained by the county-level unit of analysis in the power outage data. The variables used to compute each SVI theme are listed in Fig. 1.

We gather additional data to control for the severity of the storm and other characteristics of counties that are likely to be correlated with outage duration. We compute the maximum number of households that experience an outage in a county for a given storm to control for the direct impact of a hurricane on the local power grid. Data on hurricane wind speed is collected from the National Hurricane Center (NHC) (41). NHC reports the track of each hurricane and categorizes the maximum sustained wind speed in a location according to three categories (34–49 knots, 50–63 knots, and 64+ knots). We also control for the risk of flooding by incorporating the First Street Foundation (FSF) Flooding Risk data, which rates counties on a 1–10 scale based on the risk of future floods (42). Our models further control for a series of county-level characteristics, including size and population and also whether the county is served primarily by investor-owned utilities (IOUs).

**Fig. 2.**
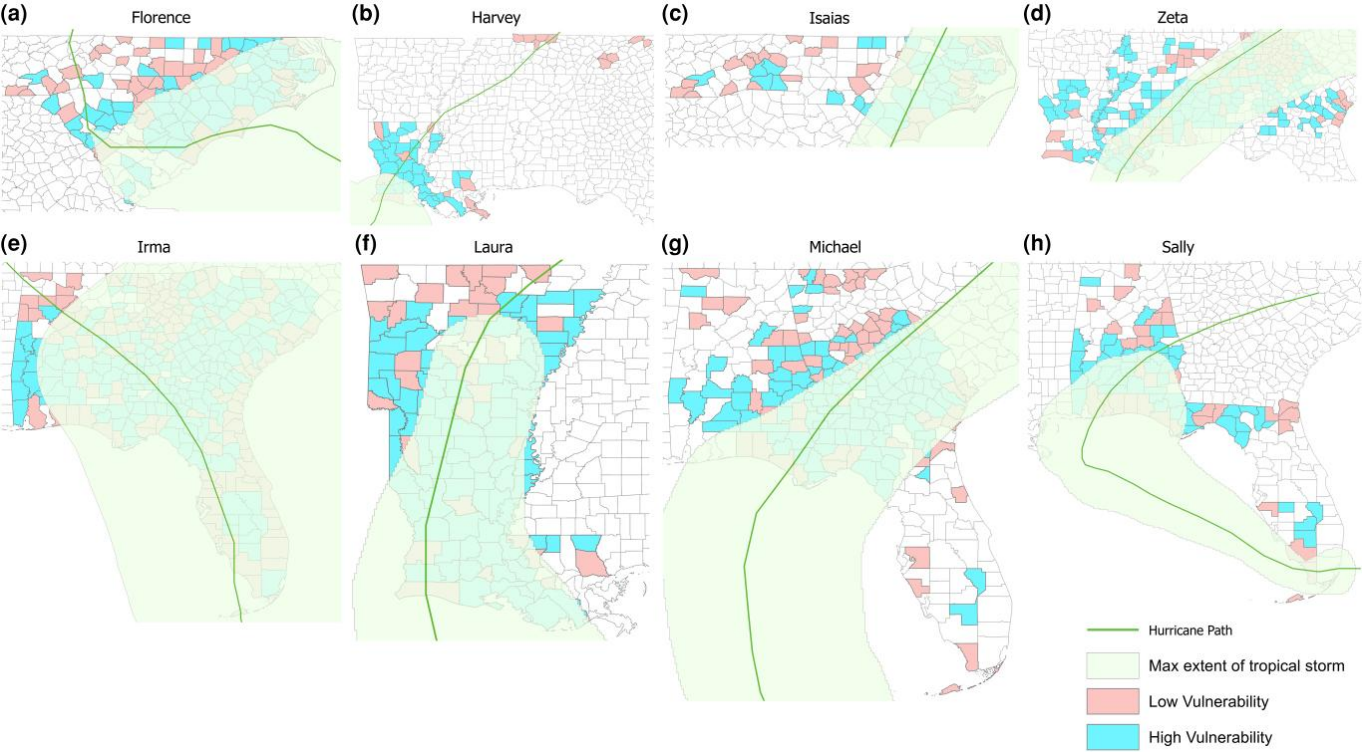
a)–h) Storm paths and affected counties for the eight hurricanes. Green line: hurricane path. Shaded green: range of sustained wind speed greater than 34 knots (tropical storm wind level). Blue: high overall social vulnerability (compared with average level of 0.726) counties. Red: low overall social vulnerability (compared with average level) counties.

Figure 2 presents the paths of each of the eight hurricanes and the regions for which the maximum sustained wind speed exceeded 34 knots (tropical storm level), as well as as the overall social vulnerability of the affected counties, where high vulnerability counties have above-average overall social vulnerability (above 0.726) and low vulnerability counties have below-average overall social vulnerability (below 0.726) among all counties that experience at least one power outage in our sample.

## Results

**Fig. 3.**
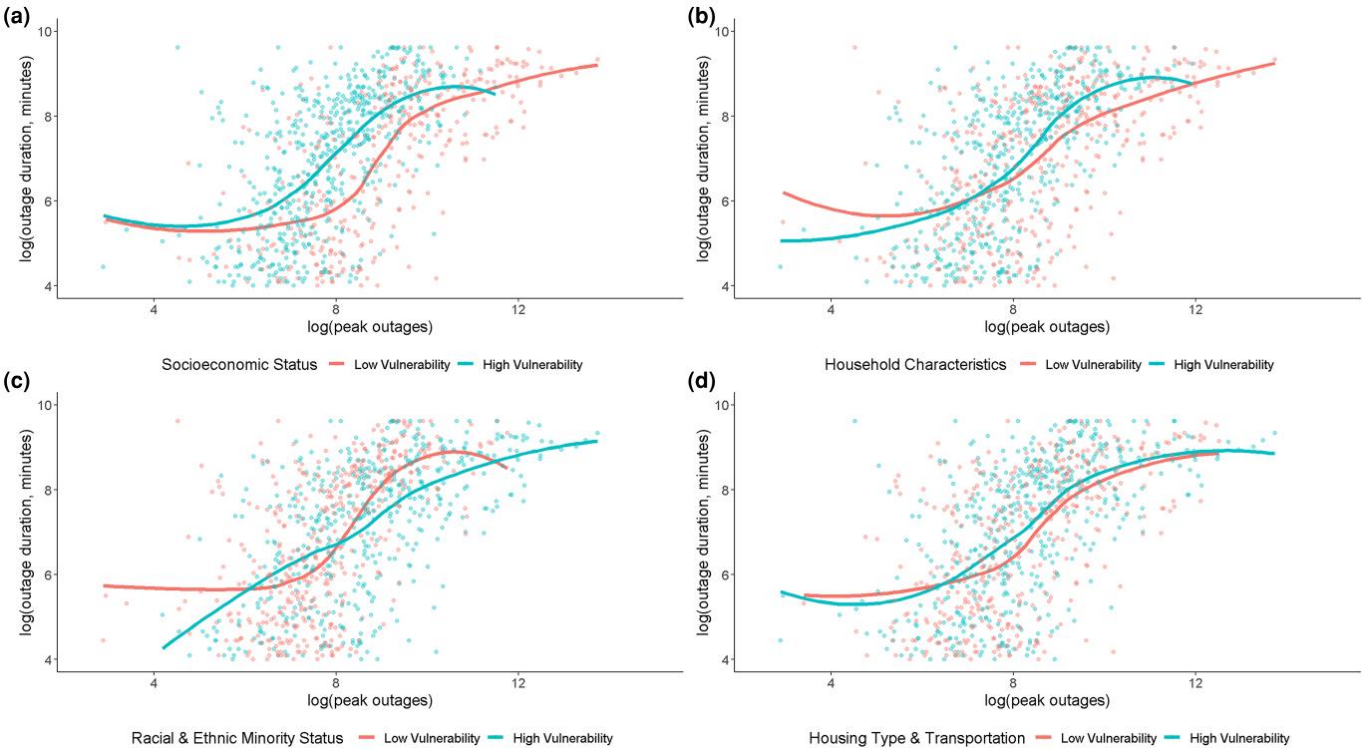
Outage duration versus the maximum number of affected customers for counties with low and high social vulnerability [a) socioeconomic vulnerability, b) household composition vulnerability, c) minority vulnerability, and d) housing type vulnerability] with local polynomial regression. Horizontal axis: log scale of the maximum number of affected customers in a county. Vertical axis: log scale of the outage duration. Blue: high social vulnerability counties. Red: low social vulnerability counties. The blue and red curves: local polynomial regression curves that show the relationship between the number of affected customers and the outage duration.

We begin our analysis by visualizing the relationship between social vulnerability and outage duration conditional on storm severity. Figure 3 plots outage duration versus the peak number of affected customers for each county and storm. Both are presented in log scale in order to account for the strong right skew of the distributions. A one-unit increase in the horizontal axis is thus equivalent to approximately a doubling of the number of affected customers and a one unit increase in the vertical axis is equivalent to approximately a doubling of the outage duration. The counties are separated into two groups: low and high social vulnerability according to the four SVI themes, where high vulnerability counties (indicated in blue) have above-average SVI values for a given theme and low vulnerability counties (indicated in red) have below-average SVI values for a given theme. We also display local polynomial regression curves in order to show whether, conditional on the peak number of affected customers, average expected outage duration is higher for more vulnerable counties than for less vulnerable counties according to the four themes. If the blue line lies above the red line, this indicates that more vulnerable counties experience longer duration outages, on average.

The plots in Fig. 3 provide preliminary evidence that socioeconomic vulnerability is correlated with longer outage recovery duration, as indicated by the wide gap between the blue and red lines in the top-left panel. For example, outages affecting 3,000 customers (i.e. 8 on the horizontal axis) last for 338 min (5.824 on the vertical axis) in a low socioeconomic vulnerability county versus 1,269 min (7.146 on the vertical axis) in a high socioeconomic vulnerability county. The other three themes, in contrast, display smaller magnitude or less consistent relationships between socioeconomic vulnerability and expected outage duration.

While the local polynomial regression curve in the top-left panel of Fig. 3 offers suggestive support of the hypothesis that socioeconomic vulnerability is correlated with prolonged outages, concerns related to unobserved heterogeneity (e.g. more vulnerable communities could experience stronger storms, on average) and spatial dependence (e.g. neighboring counties are likely to have outages of similar duration) requires a statistical model to address.

We next present the results of a series of spatial durbin models (SDMs) in order to evaluate the claim that more socioeconomically vulnerable communities experience longer outage duration, conditional on the impact of the storm itself. The SDM is a linear regression model that includes controls for the spatial lag of both the dependent and independent variables in the model in order to return an unbiased estimate of the impact of the covariates of interest (43).

We estimate the following model in our baseline specification of the SDM:


(1) $$y_{i,s}=\rho W_{i}y_{s}+X_{i,s}\beta +\theta W_{i}X_{s}+\alpha_{s}+\alpha_{st}+\epsilon ,$$

$y_{i,s}$ is the logarithm of outage duration for county *i* in storm *s*. $y_{s}$ represents the vector of outage durations for all counties for storm *s*. $W_{i}$ is a spatial weight matrix associated with county *i*. $W_{i}y_{s}$ is thus a weighted average of the outage durations among county *i*’s spatial neighbors for storm *s*. $X_{i,s}$ is a matrix of independent variables for each county-storm, including: (1) the four SVI themes, (2) logarithm of the peak number of affected customers, (3) logarithm of the county population, (4) logarithm of the county size in square-miles, (5) maximum wind speed, (6) flooding risk score, and (7) ratio of customer served by IOUs versus non-IOUs. We log-transform outage duration, the peak number of affected customers, county population, and county size in order to address both the fact that the variables are constrained to have positive values and the potential for influential outliers driven by the strong right skew in each of the variables [see, e.g. (44), p. 192–3]. $X_{s}$ is a matrix of independent variables for all counties affected by a storm. $W_{i}X_{s}$ is a weighted average of the independent variables for county *i*’s spatial neighbors. β is a vector of coefficents associated with $X_{i,s}$. $\alpha_{s}$ and $\alpha_{st}$ are storm and state fixed effects, respectively. ρ and θ characterize the strength of the spatial correlation with respect to the dependent and independent variables, respectively. ϵ is the random error term.

The regression results for the baseline specification are presented in Table 3. The first column reports the results of an SDM, referred as Model 1, in which the log of duration of power outage is regressed on each of the four SVI themes and the various control variables. The minimum-duration outage included in this analysis is 60 min, consistent with the definition of an emergency situation in (38). We find a statistically significant relationship between socioeconomic vulnerability and outage duration in a county. None of the other SVI themes exhibit a significant relationship with outage duration. ρ represents the extent of spatial dependence in the dependent variable, which is large and significant. Other covariates in the model have the expected sign and significance level. For example, an increase in the peak number of outages predicts a statistically significant increase in outage duration. The results are also consistent with standard restoration routines that are based on the desire to recover as many households as quickly as possible. Counties with higher population density, i.e. more population for a given county size, tend to have significantly shorter average outage duration than those with lower population density. We present results that include all the independent variables in the model and their spatial lags in the online supplementary Table S1.

**Table 3.** Spatial regression model results: baseline specification.

	Dependent variable:			
	log(outage duration)			
	SDM (Model 1)	MLR	SEM	SAR
log(peak outages)	0.350${}^{a}$	0.715${}^{a}$	0.416${}^{a}$	0.444${}^{a}$
	(0.035)	(0.036)	(0.035)	(0.032)
log(population)	− 0.317${}^{a}$	− 0.576${}^{a}$	− 0.355${}^{a}$	− 0.371${}^{a}$
	(0.048)	(0.050)	(0.049)	(0.042)
log(county size)	0.144	0.064	0.108	0.075
	(0.077)	(0.089)	(0.081)	(0.074)
Socioeconomic vul.	0.614${}^{a}$	0.493${}^{b}$	0.503${}^{a}$	0.504${}^{a}$
	(0.218)	(0.264)	(0.228)	(0.218)
Household characteristics vul.	− 0.202	− 0.184	− 0.127	$-0.221^{b}$
	(0.120)	(0.146)	(0.124)	(0.121)
Racial and ethnic minority status vul.	− 0.137	0.021	− 0.108	− 0.081
	(0.169)	(0.199)	(0.183)	(0.165)
Housing type and transportation vul.	− 0.059	− 0.024	− 0.084	− 0.063
	(0.131)	(0.153)	(0.138)	(0.126)
ρ	0.381${}^{a}$			0.502${}^{a}$
	(0.036)			(0.028)
λ			0.530${}^{a}$	
			(0.032)	
Socioeconomic vulnerability, average direct impact	0.589	0.493	0.503	0.548
Socioeconomic vulnerability, average 1 decile duration change (min)	170.690	135.449	112.237	144.021
LM lag test statistics		233.736${}^{a}$		
LM error test statistics		95.219${}^{a}$		
Log likelihood	− 975.870		− 1073.644	− 1018.365
LR test statistics			195.55${}^{a}$	84.99${}^{a}$
Minimum-length outage (min)	60	60	60	60
Minimum sustained wind swath (knots)	0	0	0	0
Observations	862	862	862	862

*Note*: ${}^{a}P<0.05$. ${}^{b}P<0.1$.

Other controls: IOU customer ratio, flood risk index, wind speed, state and storm dummy variables (see online supplementary material note 1).

Interpretation of the coefficients of an SDM is complicated by endogenous feedback loops among spatial neighbors. An increase in socioeconomic vulnerability in a focal county, e.g. has a first-order effect of increasing the expected outage duration of the focal county but also a second-order effect whereby it changes the expected duration in a neighboring county which feeds back into the expected duration of the focal county. We report the average direct impact implied by the model, which is the average change in the logarithm of duration in a focal county resulting from a change in the socioeconomic vulnerability of that focal county, accounting for both first-order and higher-order effects. The average direct impact is thus similar to the interpretation of a slope parameter in a classic linear regression model. Based on the coefficients in Model 1, the average direct impact of socioeconomic vulnerability is 0.589. This implies that a one-decile change in the variable, e.g. an increase from 0.7 to 0.8, produces a $e^{0.0589}-1=6.1\%$ average increase in outage duration in the focal county. (See online supplementary material note 4 for additional details on this computation.)

We next describe this impact in terms of the change in the expected outage duration. To do this, we compute the average change in outage duration conditional on a one-decile change in the socioeconomic vulnerability. Specifically, we create one synthetic dataset per county-storm in which socioeconomic vulnerability is increased by 0.1 in a focal county and kept constant in all other counties. Then, we estimate the change in predicted outage duration for the focal county using the coefficients from Model 1 for each synthetic dataset. We find that Model 1 implies that a one-decile change in socioeconomic vulnerability produces an average increase in outage duration of approximately 170 min.

An SDM is appropriate in cases where the outcome in a focal county may be impacted by outcomes or characteristics of other nearby counties. Alternative approaches to spatial regression include multiple linear regression (MLR), spatial error models (SEM), and spatial autoregression models (SAR). Each of these alternative approaches can be viewed as a restriction on the parameters estimated in the SDM. The second, third, and fourth columns of the table report results from these other approaches. The second column reports results from an MLR. This specification assumes that there is no spatial correlation among counties, i.e. ρ=0, θ=0, and ϵ is not spatially autocorrelated. We use a Lagrange multiplier test to evaluate the possibility of spatial autocorrelation, which indicates that the MLR is misspecified at the P=0.05 level. The third column reports results from an SEM, which assumes spatial correlation in errors, but not the independent or dependent variables. The fourth column reports results from an SAR, which assumes spatial correlation in the independent variable, but not the dependent variables. Both the SAR and SEM model are rejected in favor of the SDM model at the P=0.05 level by a likelihood ratio test, which explains why the SDM is our preferred model.^a^ That being said, the broad consistency of the effect of socioeconomic vulnerability—the coefficient value ranges from 0.493 in the MLR to 0.614 in the SDM—on expected outage duration provides confidence that the observed result is not an artifact of particular modeling choices.

**Fig. 4.**
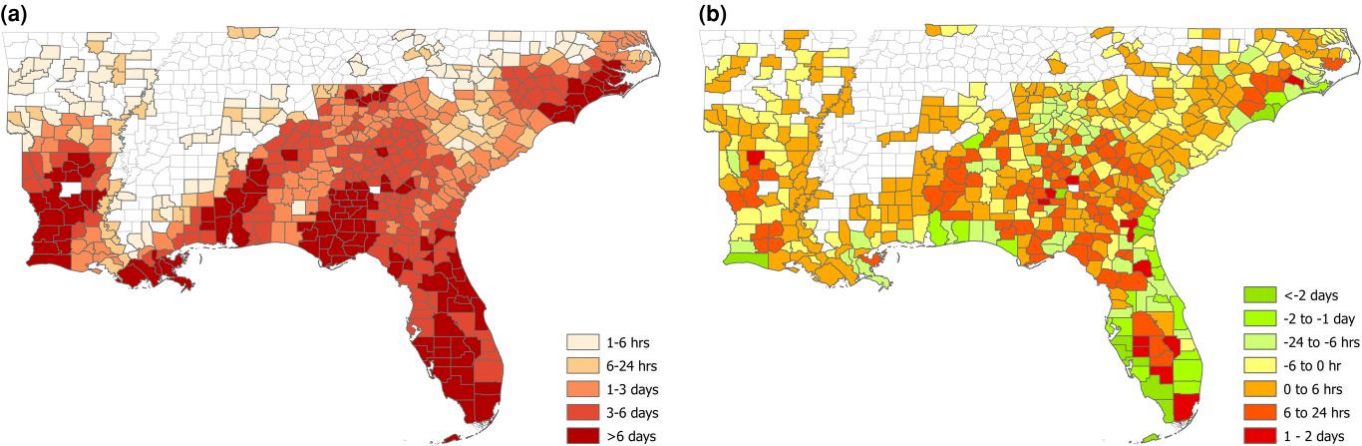
a) Geospatial plot of the total observed duration of power outages summed over the eight storms. b) The difference between the total predicted duration from Model 1 and the total predicted duration holding socioeconomic vulnerability at its average value of 0.718.

In Fig. 4, we illustrate the spatial distribution of the effect identified in Model 1. In the left panel, we plot the total observed outage duration for each county, summed across the eight storms. The counties shaded in dark red indicate locations with longer total outage duration than those shaded in lighter red. The right panel shows the difference between the expected total outage duration predicted using Model 1 and the expected total outage duration when all counties are held constant at the average level of socioeconomic vulnerability among affected counties (i.e. 0.718). For the counties in red, the expected total outage duration when socioeconomic vulnerability is held constant is shorter than than the expected duration predicted by Model 1. The average level of socioeconomic vulnerability for 101 counties that experience at least 6 additional hours of expected power outages is 0.91. For counties in green, the expected total outage duration when socioeconomic vulnerability is held constant is longer than the expected duration predicted by Model 1. The average level of socioeconomic vulnerability for the 89 counties that experience at least 6 fewer hours of expected power outages is 0.38. The 332 counties that are colored yellow or orange in the plot experience between 6 more and 6 fewer hours of power outages. Their average socioeconomic vulnerability is 0.75. (See online supplementary material note 5 for additional details.)

We next demonstrate the robustness of our results in a series of models with varying county-level selection criteria and covariates included in the model. The results are presented in Table 4. In Models 2 and 3, we reduce the minimum outage duration criterion from 60 to 5 min and 30 min, respectively. Model 2 increases the sample size by 242 observations. Model 3 increases the sample size by 81 observations. The coefficient on socioeconomic vulnerability remains significantly greater than 0 across both models. In Model 4, we include only counties that experience maximum sustained wind speeds of 34 knots or higher and are thus comparable in terms of their risk for a storm-caused power outage. This restriction decreases the sample size by 171 observations. The estimated effect of socioeconomic vulnerability remains positive and significant.

**Table 4.** Spatial regression model results: alternative specifications.

	Dependent variable:						
	log(outage duration)						
	Model 2	Model 3	Model 4	Model 5	Model 6	Model 7	Model 8
log(peak outages)	0.495${}^{a}$	0.391${}^{a}$	0.407${}^{a}$	0.358${}^{a}$	0.349${}^{a}$	0.350${}^{a}$	0.348${}^{a}$
	(0.039)	(0.036)	(0.037)	(0.035)	(0.035)	(0.035)	(0.035)
log(population)	− 0.499${}^{a}$	− 0.393${}^{a}$	− 0.396${}^{a}$	− 0.355${}^{a}$	− 0.389${}^{a}$	− 0.387${}^{a}$	− 0.376${}^{a}$
	(0.057)	(0.050)	(0.050)	(0.044)	(0.042)	(0.042)	(0.042)
log(county size)	0.186	0.185${}^{a}$	0.154${}^{a}$	0.147	0.177${}^{a}$	0.172${}^{a}$	0.159${}^{a}$
	(0.099)	(0.084)	(0.075)	(0.077)	(0.077)	(0.077)	(0.077)
Socioeconomic vul.	0.684${}^{a}$	0.540${}^{a}$	0.504${}^{a}$	0.386${}^{a}$			
	(0.267)	(0.231)	(0.220)	(0.157)			
Household characteristics vul.	− 0.294	− 0.193	− 0.087		− 0.037		
	(0.152)	(0.129)	(0.120)		(0.107)		
Racial and ethnic minority status vul.	− 0.064	− 0.126	− 0.473${}^{a}$			0.010	
	(0.211)	(0.180)	(0.178)			(0.161)	
Housing type and transportation vul.	− 0.138	− 0.028	0.031				0.109
	(0.161)	(0.139)	(0.130)				(0.104)
ρ	0.400${}^{a}$	0.406${}^{a}$	0.488${}^{a}$	0.385${}^{a}$	0.385${}^{a}$	0.386${}^{a}$	0.383${}^{a}$
	(0.032)	(0.034)	(0.039)	(0.036)	(0.036)	(0.036)	(0.036)
Average direct socioeconomic impact	0.787	0.596	0.549	0.384			
Minimum-length outage (min)	5	30	60	60	60	60	60
Minimum sustained wind swath (knots)	0	0	34	0	0	0	0
Observations	1,104	943	691	862	862	862	862
Log likelihood	− 1,633.431	− 1,173.041	− 718.029	− 981.319	− 980.965	− 981.164	− 979.025

*Note*: Other controls: IOU customer ratio, flood risk index, wind speed, state, and storm dummy variables (see online supplementary material note 1 and Table S1 for complete results).

${}^{a}P<0.05$ .

In Models 5 through 8, we estimate the impact of each SVI theme individually. When socioeconomic vulnerability is the only SVI theme included in the model, the coefficient value declines to 0.386 but remains significantly greater than zero. This attenuation is explained by the positive correlation between socioeconomic vulnerability and the other three SVI themes, each that have an insignificantly negative impact on expected outage duration in Model 1 in Table 3. When the other SVI themes are included individually, the estimated coefficients are not statistically different from zero. These results, taken together, present strong, robust evidence of the relationship between socioeconomic vulnerability and outage duration. Further, they do not indicate a relationship between the other SVI themes and outage duration.

In our final set of analyses, we explore storm-level heterogeneity in the effect of socioeconomic vulnerability on expected outage duration. Our results are presented in Table 5 (see online supplementary material note 2 and Table S2 for complete results). The results are ambiguous. On the one hand, the coefficient on socioeconomic vulnerability is positive in seven of the eight storms. Further, among the 7 storms, just one pair (Michael and Irma) has coefficient values that differ at the P=0.05 level. As a result, the storm-level analysis offers confidence that the SDM results reported in the first column of Table 3 are not being driven exclusively by a single storm. On the other hand, just two of the effects are significantly different from zero and the effect sizes range from −0.672 (Isaias) to 1.394 (Michael). The variability in the coefficient levels together with the wide 95% confidence intervals thus suggest the possibility of storm-level heterogeneity that is worth exploring in future research.

**Table 5.** Spatial regression model results: storm-varying effects.

	Florence	Harvey	Irma	Isaias	Laura	Michael	Sally	Zeta	Gulf	Non-Gulf
Socioeconomic vul.	0.498	1.147	0.342	− 0.672	1.026${}^{a}$	1.394${}^{b}$	1.334${}^{b}$	0.439	0.650${}^{b}$	0.021
Standard error	0.533	0.964	0.273	0.643	0.572	0.377	0.563	0.361	0.221	0.438

*Note*: Non-Gulf hurricanes includes Florence and Isaias.

${}^{a}P<0.1$ . ${}^{b}P<0.05$.

One possible source of heterogeneity indicated in Table 5 is the location of hurricane landfall. Note that we observe the strongest statistical relationships between socioeconomic vulnerability and outage duration for Laura, Michael, and Sally, three hurricanes that made landfall over the Gulf coast. In the final two columns of the table, we report results from a model that estimates different coefficients on socioeconomic vulnerability for hurricanes that made landfall over the Gulf coast (Harvey, Irma, Laura, Michael, Sally, and Zeta) versus those that made landfall over the Atlantic coast (Florence and Isaias). We observe that the effect for Gulf hurricanes (0.650) is in line with our estimate based on the entire sample (0.614), whereas the non-Gulf hurricanes have a smaller coefficient value (0.021). However, coefficient values on Gulf and non-Gulf hurricanes are not statistically different from each other at the P=0.05 significance level (see online supplementary Table S3 for complete results).

## Discussion

Using data on large-scale power outages induced by eight major hurricanes that affected nine states in the Southeast United States, our analysis finds that communities with more socioeconomic vulnerability experience longer-duration power outages after controlling for the direct impact of the storm. In contrast, the other three SVI themes—Household characteristics vulnerability, Racial & ethnic minority status vulnerability and Housing type & transportation vulnerability—do not exhibit a statistically significant relationship with outage duration. The affected service regions across the hurricanes account for roughly one-tenth of the United States in terms of size and one-twelfth in terms of population. Thus, we feel confident that our results are not driven by a single storm, state, or utility.

Our results point to the necessity of reexamining commonly used policies for disaster recovery, infrastructure investment, and poststorm resource allocation. Adjusting storm-recovery priorities so that communities for whom prolonged failures are more disruptive are recovered relatively sooner is one possible intervention, but also one that could come at the cost of longer duration outages overall. Alternative approaches include making the grid more robust and households in economically and socially vulnerable communities more resilient to storm-caused power failures (45). Additional investment in energy infrastructure and renewable energy in these communities may reduce the likelihood that outages occur or permit households access to other resources when power outages do happen. These interventions are likely to require coordination among policymakers, regulators, utilities, and communities, as practices related to investment, pricing, and outage recovery tend to be highly regulated (46, 47).

The study also speaks to the need for more detailed and granular data on power outages to be made available over large spatiotemporal scales for both researchers and practitioners. In particular, more granular data on communities and households measured over extended time periods is necessary to dig deeper into the mechanisms that lead socioeconomically vulnerable communities to experience longer outages, to analyze the negative impact of these outages on economically and socially vulnerable households, and to design appropriate policy interventions. Standard models of the negative impact of natural disasters evaluate risk as a function of (1) the likelihood of a threat occurring, (2) the extent of harm conditional on an event occurring, and (3) the ability for a household to diminish the effect of the hazard (25). While our study yields additional insight into (2), we look forward to future studies that also integrate data on (1) and (3) in order to better model the distribution of risk conditional on economic and social vulnerability. Furthermore, there is likely to be within-county heterogeneity in both storm impact and economic and social vulnerability that is masked by the county-level geographic unit of analysis in our dataset. This lack of granularity in the power outage data also limits our ability to make strong claims about the reasons we do not observe correlations between the other three SVI themes and outage duration. We encourage readers to interpret these imprecisely estimated null effects as motivation to conduct additional studies with better data, rather than as well-estimated zeroes.

To the extent there is a tradeoff between recovering those communities who can least afford extended power outages and recovering as many households as possible, these data would also permit researchers and policymakers to better quantify those tradeoffs and then design interventions that achieve an optimal balance. Even better would be for decision makers to integrate real-time information about the vulnerability of households when prioritizing recovery decisions. These data would permit utilities to allocate limited resources in a manner that minimizes economic cost—e.g. by prioritizing smaller communities that lack access to backup power and the resources to evacuate prior to a storm over larger ones that have backup power or who have evacuated—rather than seeking to minimizing the aggregate outage length.

Given the growing frequency and severity of damaging weather events resulting from climate change (48, 49), we expect that the issues identified in our analysis will continue to grow in importance, especially in light of the recent policy emphasis on reducing the differential climate impact on disadvantaged communities (8–11). Re-calibrating infrastructure investment and routines surrounding disaster recovery to ensure that increasingly damaging storms do not further exacerbate other sources of community vulnerability is a necessary next step and requires an integrated approach in which policymakers, regulators, utilities, and communities work together to ensure that the most economically and socially vulnerable households enjoy energy equity and are not the ones consistently with the longest spells without power.

## Supplementary Material

pgad295_Supplementary_Data

## Data Availability

All publicly available data and all code used in the study to produce the tables and figures will be made available as part of a complete set of replication files. The power outage data is a proprietary dataset entitled “Atlantic Hurricanes 2017 to 2020—City By Utility Datasets” purchased from PowerOutage.com. With the replication files, any researcher will be able to purchase the data from the vendor and replicate our analysis in its entirety. The list of affected states and corresponding period of time in our dataset follows: Alabama: 2017 August 30–2017 October 14; 2018 October 1–2018 October 30; 2020 September 11–2020 October 10; 2020 October 24–2020 November 20 Arkansas: 2020 August 20–2020 September 15 Florida: 2017 August 30–2017 October 14; 2018 October 1–2018 October 30; 2020 September 11–2020 October 10 Georgia: 2017 August 30–2017 October 14; 2018 October 1–2018 October 30; 2020 October 24–2020 November 20 Louisiana: 2017 August 17–2017 September 22; 2020 August 20–2020 September 15; 2020 October 24–2020 November 20 Mississippi: 2020 October 24–2020 November 20 North Carolina: 2018 August 30–2018 October 3; 2020 July 30–2020 August 30 South Carolina: 2017 August 30–2017 October 14; 2018 August 30–2018 October 3 Tennessee: 2017 August 17–2017 September 22

## Footnotes

a These specification tests are presented in Table 3 with additional detail on each test included in the online supplementary material.

## References

[1] The White House . 2021. Justice40. Washington (DC), USA. [accessed 2023 Sep 18]. https://www.whitehouse.gov/environmentaljustice/justice40/.

[2] U.S. Department of Energy . 2021. Justice40 initiative. Department of Energy. [accessed 2023 Sep 18]. https://www.energy.gov/diversity/justice40-initiative.

[3] Mitsova D , Esnard AM, Sapat A, Lai BS. 2018. Socioeconomic vulnerability and electric power restoration timelines in Florida: the case of Hurricane Irma. Nat Hazards. 94(2):689–709.

[4] Ulak MB , Kocatepe A, Konila Sriram LM, Ozguven EE, Arghandeh R. 2018. Assessment of the hurricane-induced power outages from a demographic, socioeconomic, and transportation perspective. Nat Hazards. 92(3):1489–1508.

[5] Flores AB , Collins TW, Grineski SE, Chakraborty J. 2020. Social vulnerability to Hurricane Harvey: unmet needs and adverse event experiences in Greater Houston, Texas. Int J Disaster Risk Reduct. 46:101521.

[6] Sotolongo M , Kuhl L, Baker SH. 2021. Using environmental justice to inform disaster recovery: vulnerability and electricity restoration in Puerto Rico. Environ Sci Policy. 122:59–71.

[7] Best K , *et al*. 2023. Spatial regression identifies socioeconomic inequality in multi-stage power outage recovery after Hurricane Isaac. Nat Hazards. 117(1):851–873.

[8] National Research Council . 2012. Disaster resilience: a national imperative. [accessed 2023 Sep 18]. https://nap.nationalacademies.org/read/13457/chapter/1.

[9] National Research Council . 2015. Developing a framework for measuring community resilience: summary of a workshop. [accessed 2023 Sep 18]. https://nap.nationalacademies.org/read/20672/chapter/1.

[10] National Academies of Sciences, Engineering, and Medicine . 2019. Building and measuring community resilience: actions for communities and the Gulf Research Program. [accessed 2023 Sep 18]. https://nap.nationalacademies.org/read/25383/chapter/1.

[11] National Academies of Sciences, Engineering, and Medicine . 2022. Investing in resilient infrastructure in the Gulf of Mexico: proceedings of a workshop. [accessed 2023 Sep 18]. https://nap.nationalacademies.org/read/26559/chapter/1.

[12] Edison Electric Institute . 2016. Understanding the electric power industry’s response and restoration process. Edison Electric Institute. Technical report.

[13] American Public Power Association . 2018. Restoration best practices guidebook. American Public Power Association. Technical report.

[14] Tormos-Aponte F , García-López G, Painter MA. 2021. Energy inequality and clientelism in the wake of disasters: from colorblind to affirmative power restoration. Energy Policy. 158:112550.

[15] Huang CC , Taylor R. 2019. Any federal infrastructure package should boost investment in low-income communities. Washington (DC): Center for Budget and Policy Priorities. Technical report. https://www.cbpp.org/sites/default/files/atoms/files/4-4-19bud.pdf.

[16] Jones SH , Armanios DE. 2020. Methodological framework and feasibility study to assess social equity impacts of the built environment. J Constr Eng Manag. 146(11):05020016.

[17] Reta M , Gout E. 2021. Advancing equity through grid modernization. [accessed 2023 Sep 18]. https://www.americanprogress.org/article/advancing-equity-grid-modernization/.

[18] Fothergill A , Peek LA. 2004. Poverty and disasters in the United States: a review of recent sociological findings. Nat Hazards. 32(1):89–110.

[19] Thomas K , *et al*. 2019. Explaining differential vulnerability to climate change: a social science review. WIREs Clim Change. 10(2):e565. 10.1002/wcc.565 PMC6472565 31007726

[20] Roman MO , *et al*. 2019. Satellite-based assessment of electricity restoration efforts in Puerto Rico after Hurricane Maria. PLoS ONE. 14(6):e0218883. 31251791 10.1371/journal.pone.0218883 PMC6599127

[21] Afsharinejad AH , Ji C, Wilcox R. 2021. Large-scale data analytics for resilient recovery services from power failures. Joule. 5(9):2504–2520.

[22] Flanagan B , Hallisey EJ, Adams E, Lavery A. 2018. Measuring community vulnerability to natural and anthropogenic hazards: the centers for disease control and prevention’s social vulnerability index. J Environ Health. 80(10):34–36. PMC7179070 32327766

[23] Eisenman DP , *et al*. 2009. Variations in disaster preparedness by mental health, perceived general health, and disability status. Disaster Med Public Health Prep. 3(1):33–41. 19293742 10.1097/DMP.0b013e318193be89

[24] Hong B , Bonczak BJ, Gupta A, Kontokosta CE. 2021. Measuring inequality in community resilience to natural disasters using large-scale mobility data. Nat Commun. 12(1):1870. 33767142 10.1038/s41467-021-22160-w PMC7994553

[25] Flanagan BE , Gregory EW, Hallisey EJ, Heitgerd JL, Lewis B. 2011. A social vulnerability index for disaster management. J Homel Secur Emerg Manag. 8(1):Article 3.

[26] Centers for Disease Control and Prevention . 2022. CDC/ATSDR SVI 2020 documentation. [accessed 2023 Sep 18]. https://www.atsdr.cdc.gov/placeandhealth/svi/documentation/pdf/SVI2020Documentation_08.05.22.pdf.

[27] GeoPlatform . 2021. Climate and economic justice screening tool. [accessed 2023 Sep 18]. https://screeningtool.geoplatform.gov.

[28] Lee DC , *et al*. 2016. Geographic distribution of disaster-specific emergency department use after Hurricane Sandy in New York City. Disaster Med Public Health Prep. 10(3):351–361. 26857616 10.1017/dmp.2015.190 PMC7112993

[29] Skarha J , *et al*. 2021. Association of power outage with mortality and hospitalizations among Florida nursing home residents after Hurricane Irma. JAMA Heal Forum. 2(11):e213900. 10.1001/jamahealthforum.2021.3900 PMC8796882 35977265

[30] Kohn S , *et al*. 2012. Personal disaster preparedness: an integrative review of the literature. Disaster Med Public Health Prep. 6(3):217–231. 23077264 10.1001/dmp.2012.47

[31] Lievanos RS , Horne C. 2017. Unequal resilience: the duration of electricity outages. Energy Policy. 108:201–211.

[32] Coleman N , *et al*. 2023. Energy inequality in climate hazards: empirical evidence of social and spatial disparities in managed and hazard-induced power outages. Sustain Cities Soc. 92:104491.

[33] Voss PR , Long DD, Hammer RB, Friedman S. 2006. County child poverty rates in the US: a spatial regression approach. Popul Res Policy Rev. 25(4):369–391.

[34] Logan JR , Xu Z. 2015. Vulnerability to hurricane damage on the U.S. Gulf Coast since 1950. Geogr Rev. 105(2):133–155. 25926706 10.1111/j.1931-0846.2014.12064.x PMC4410365

[35] [dataset]* PowerOutages . 2021. Power outage records of the Southeast US. [accessed 2023 September 18]. https://poweroutage.us/products.

[36] Federal Emergency Management Agency . 2022. Fema major disaster hurricane declaration. [accessed 2023 Sep 18]. https://www.fema.gov/disaster/declarations.

[37] National Oceanic and Atmospheric Administration . 2022. NOAA the costliest U.S. tropical cyclones. [accessed 2023 September 18]. https://www.ncei.noaa.gov/access/billions/dcmi.pdf.

[38] U.S. Department of Energy . 2021. OE-417: electric emergency incident and disturbance report. [accessed 2023 Sep 18]. https://www.oe.netl.doe.gov/docs/OE417_Form_Instructions_05312021.pdf.

[39] [dataset]* Centers for Disease Control and Prevention/ Agency for Toxic Substances and Disease Registry/ Geospatial Research, Analysis, and Services Program . 2022. CDC/ATSDR Social vulnerability index 2018 database, USA. [accessed 2023 Sep 18]. https://www.atsdr.cdc.gov/placeandhealth/svi/data_documentation_download.html.

[40] Do V , *et al*. 2023. Spatiotemporal distribution of power outages with climate events and social vulnerability in the USA. Nat Commun. 14(1):2470. 37120649 10.1038/s41467-023-38084-6 PMC10147900

[41] [dataset]* National Hurricane Center . 2022. Atlantic hurricane best track database. [accessed 2023 September 18]. https://www.nhc.noaa.gov/data/tcr/index.php.

[42] [dataset]* First Street Foundation . 2022. First street foundation flood factor data. [accessed 2023 September 18]. https://firststreet.org/risk-factor/flood-factor/.

[43] LeSage JP , Pace RK. 2009. Introduction to spatial econometrics. Statistics, textbooks and monographs. Boca Raton: CRC Press.

[44] Wooldridge JM . 2013. Introductory econometrics: a modern approach. 5th ed. Mason (OH): South-Western Cengage Learning.

[45] Brockway AM , Conde J, Callaway D. 2021. Inequitable access to distributed energy resources due to grid infrastructure limits in California. Nat Energy. 6(9):892–903.

[46] Lazar J . 2016. Electricity regulation in the US: a guide. Regulatory Assistance Project.

[47] Oppenheim J . 2016. The United States regulatory compact and energy poverty. Energy Res Soc Sci. 18:96–108.

[48] Seneviratne SI , *et al*. 2021. Weather and climate extreme events in a changing climate. Cambridge, UK: Cambridge University Press. p. 1513–1766.

[49] Emanuel K . 2021. Atlantic tropical cyclones downscaled from climate reanalyses show increasing activity over past 150 years. Nat Commun. 12(1):1–8. 34857770 10.1038/s41467-021-27364-8 PMC8639808

