# Supplementary material for: Socioeconomic vulnerability and differential impact of severe weather-induced power outages
Source: PNAS Nexus. 2023 Oct 3;2(10):pgad295. doi: 10.1093/pnasnexus/pgad295 (PMC10547019; doi:10.1093/pnasnexus/pgad295)
Supplement: pgad295_Supplementary_Data [file pgad295_supplementary_data.zip › PNASNEXUS-PNASNEXUS-2023-00296R-s01.pdf]

Supporting Information for

Socioeconomic vulnerability and differential impact of severe weather-induced power outages

Scott C. Ganz, Chenghao Duan, Chuanyi Ji

To whom correspondence should be addressed. E-mail: scott.ganz@georgetown.edu

This PDF file includes:

Supporting text
Tables S1 to S3
SI References

Supporting Information Text

Supplementary information note 1: SDM results with all independent variables. We present the Spatial Durbin models' (SDM) results with all variables. The results are shown in Table S1. In our model, variables labeled with "fixed effect" are categorical variables for states, storms, and maximum sustained wind speeds (wind swath); variables labeled with " spatial lag" are the spatial lag terms for the independent variables, which correspond to the θ in the SDM formula; "ρ" is the spatial lag term of the dependent variable. The tables are from R code for reproduction of our results.

Supplementary information note 2: SDM with storm-level heterogeneity. This supplemental note provides additional information on the alternative spatial Durbin models in the main text. We test the possibility of storm-level heterogeneity in relationship between socioeconomic vulnerability and expected outage duration. We use the notation below to represents the socioeconomic vulnerability $SVI1$, of each county i affected by storm s,

$$SVI1_i^s = SVI1_i \cdot I\{\text{storm} = s\}$$

This model thus isolates the effect of socioeconomic vulnerability within each storm event. The results are presented in Table S2.

Further, we separate the hurricanes into 2 groups: gulf and non-gulf hurricanes, depending on whether the hurricane affected the Gulf of Mexico. The non-gulf hurricanes, denoted with a dummy variable $g = 0$, include the Hurricane Florence and the Hurricane Isaias, and the gulf hurricanes, for which $g = 1$, include the others. The results are presented in Table S3

$$SVI1_i^{\text{gulf}} = SVI1_i \cdot I\{g = 1\}$$
$$SVI1_i^{\text{non-gulf}} = SVI1_i \cdot I\{g = 0\}$$

Supplementary information note 3: Statistical tests for alternative spatial models. In this note, we explain the statistical tests we performed to justify the choice of Spatial Durbin Model (SDM) for the power outage dataset.

First, we test the spatial correlation in multiple linear model through the Lagrange multiplier (LM) tests: LM lag test and LM error test. The multiple linear regression (MLR) model to be tested is obtained with the same set of variables and dataset as the SDM through the ordinary least square (OLS) algorithm. MLR can be specified as a restricted case of a spatial model, with 0 coefficients for all the spatial lag terms:

$$y = X\beta + \rho W y + u$$
$$u = \lambda W \mu + e$$

where W is the spatial weight matrix, μ is the spatially uncorrelated errors, and e is a well-behaved and not autocorrelated error term.

LM lag test statistics tests for the null hypothesis with a missing spatially lagged dependent variable ($\rho = 0$), and LM error test statistics tests for no spatial autocorrelation in error ($\lambda = 0$) (1, 2). The tests statistics are obtained with spdep (version 1.2-8) package in R (3). The LM lag and error tests thus reject both of the null hypotheses, indicating the need for spatial models.

Next, we compare the performances of different spatial models, namely Spatial Autoregressive Model (SAR, only spatially lagged dependent variables), Spatial Error Model (SEM, only accounting spatial dependence in error term), and Spatial Durbin model (SDM, both spatially lagged dependent and independent variables). They are specified as followed:

$$\text{SAR: } y = \rho W y + X\beta + e$$
$$\text{SEM: } y = X\beta + \lambda W u + e$$
$$\text{SDM: } y = \rho W y + X\beta + W X\theta + e$$

We compute the log likelihood and perform pairwise likelihood ratio (LR) tests of SDM with alternative models using spatialreg packeage in R (3). The results, listed in Table 3. in main, show that the SDM outperformed the other alternative models significantly. Thus we justify our choice of the SDM for the power outage dataset.

Supplementary information note 4: Computation of average direct effects and average change in outage duration associated with a one-decile increase in socioeconomic vulnerability.. We first explain the average direct impact of socioeconomic vulnerability listed in Table 3. in main. The average direct impact of socioeconomic vulnerability refers to the average change in logarithm of outage duration from the change of socioeconomic vulnerability, which is similar with the average partial derivative for logarithm of outage duration with respect to socioeconomic vulnerability. Derived from (4), for each observation i, the direct impact of socioeconomic vulnerability $SVI1$ is given in the following form:

$$\frac{\partial y_i}{\partial SVI1_i} = S_{SVI_1}(W)_{ii}$$

where $S_{SVI_1}(W)_{ii}$ is the ith diagonal entry of matrix $S_{SVI_1}(W)$, and y_i is the logarithm of outage duration for observation i.

For different models, the matrix $S_{SVI_1}(W)$ takes different forms:

$$\begin{cases} S_{SVI_1}(W) = I_n \beta_{SVI_1} & \text{for MLR} \\ S_{SVI_1}(W) = I_n \beta_{SVI_1} & \text{for SEM} \\ S_{SVI_1}(W) = (I_n - \rho W)^{-1} I_n \beta_{SVI_1} & \text{for SAR} \\ S_{SVI_1}(W) = (I_n - \rho W)^{-1} (I_n \beta_{SVI_1} + W \theta_{SVI_1}) & \text{for SDM} \end{cases}$$

where W is the spatial weight matrix and n is the number of rows/columns of W.

The average direct impact is the average of $S_{SVI_1}(W)_{ii}$ over all observations:

$$ADI = n^{-1} tr(S_{SVI_1}(W))$$

For example, SDM has an average direct impact of 0.589, which means that on average for every 1 decile increase of socioeconomic vulnerability, the outage duration would increase by 6.06%.

The average change in expected outage duration associated with a one-decile change in socioeconomic vulnerability is estimated in the following manner: For each observation, we generate a synthetic dataset in which the socioeconomic vulnerability of that observation is increased by 0.1 while other independent variables of the observation and other observations hold the same. The outage duration for that observation is then estimated using each model and compared with the original model estimated outage duration. Estimations are performed with predict functions from corresponding model package in R. Note that the comparison is between the selected observation from original and synthetic dataset only, which does not include the indirect impacts on the neighbors. We compute the average of the differences between the predicted outcomes from the original model and the predicted outcomes in the synthetic datasets for each focal county. The average of duration changes in minutes for all the models are recorded in Table 3 in main.

Supplementary information note 5: Detail for Figure 4. In this note, we explain the Fig 4 in main. In the left panel, we illustrate the total outage duration d_j caused by all 8 hurricane events for each affected county j whose outage duration is at least 60 minutes, i.e. $d_j = \sum_s d_{j,s}$, where $d_{j,s}$ is the outage duration in county j caused by storm s. To further identify the differential impact, we compared the estimated outage duration for each observation of county-event pair with that of the counterfactual scenario for even socioeconomic vulnerability distribution, i.e. for each county j, we manually alter the socioeconomic vulnerability to the average level among all the county: $SVI1_j = S\bar{V}I1 = 0.718$, and all the other individual variables are unchanged. Given the synthetic dataset and original dataset, we used our preferred SDM Model 1 to estimate the outage duration for each observation in two datasets and compare them: $\delta \hat{d}_{j,s} = \hat{d}^o_{j,s} - \hat{d}^e_{j,s}$, where $\hat{d}^o_{j,s}$ is the estimated outage duration of county j in storm s from the original dataset and $\hat{d}^e_{j,s}$ is the estimated outage duration of county j in storm s from the synthesized dataset. The estimations are performed with predict function from spdep package in R. If $\delta \hat{d}_{j,s} > 0$, county j is relatively more vulnerable county in storm s, which took longer to recover than an average county under the same condition. Then for each county j, we consider the total difference in duration among all the hurricane events: $\delta \hat{d}_j = \sum_s \delta \hat{d}_{j,s}$, which represents a total differential impact. $\delta \hat{d}_j$ is then plotted in the right panel, where we used orange to red to represent counties with $\delta \hat{d}_j > 0$, and yellow to green for counties with $\delta \hat{d}_j \leq 0$.

Table S1. Spatial Durbin models results with all independent variables

	Dependent variable:				
	log(outage duration)				
	Model 1	Model 2	Model 3	Model 4	Model 5
log(peak outages)	0.350*	0.495*	0.391*	0.407*	0.358*
	(0.035)	(0.039)	(0.036)	(0.037)	(0.035)
log(population)	−0.317*	−0.499*	−0.393*	−0.396*	−0.355*
	(0.048)	(0.057)	(0.050)	(0.050)	(0.044)
log(county size)	0.144	0.186	0.185*	0.154*	0.147
	(0.077)	(0.099)	(0.084)	(0.075)	(0.077)
Ratio of customer served by IOU	0.079	0.021	0.099	0.152	0.056
	(0.102)	(0.123)	(0.106)	(0.104)	(0.102)
FSF flood risk score	0.012	0.005	−0.010	0.027	0.011
	(0.037)	(0.047)	(0.040)	(0.036)	(0.037)
Socioeconomic Vulnerability	0.614*	0.684*	0.540*	0.504*	0.386*
	(0.218)	(0.267)	(0.231)	(0.220)	(0.157)
Household Characteristics Vulnerability	−0.202	−0.294	−0.193	−0.087	
	(0.120)	(0.152)	(0.129)	(0.120)	
Racial & Ethnic Minority Vulnerability	−0.137	−0.064	−0.126	−0.473*	
	(0.169)	(0.211)	(0.180)	(0.178)	
Housing Type & Transportation Vulnerability	−0.059	−0.138	−0.028	0.031	
	(0.131)	(0.161)	(0.139)	(0.130)	
Arkansas, state fixed effect	−0.667	−1.566	−0.110	−0.770	−0.823
	(1.194)	(1.325)	(1.296)	(1.303)	(1.198)
Florida, state fixed effect	0.047	0.133	0.001	0.030	0.058
	(0.315)	(0.434)	(0.354)	(0.321)	(0.314)
Georgia, state fixed effect	−0.252	−0.376	−0.241	−0.425	−0.284
	(0.265)	(0.375)	(0.301)	(0.279)	(0.265)
Louisiana, state fixed effect	0.115	−1.374	0.244	−0.457	0.063
	(1.047)	(1.070)	(1.140)	(1.141)	(1.048)
Mississippi, state fixed effect	0.055	0.384	0.010	−0.196	0.019
	(0.740)	(0.763)	(0.709)	(0.630)	(0.742)
North Carolina, state fixed effect	−1.366	−1.042	−1.281	−1.129	−1.312
	(0.755)	(0.980)	(0.850)	(0.700)	(0.757)
South Carolina, state fixed effect	−1.254*	−1.373	−1.216*	−1.401*	−1.245*
	(0.504)	(0.712)	(0.565)	(0.477)	(0.506)
Tennessee state, fixed effect	0.640	0.573	0.817		0.700
	(0.407)	(0.468)	(0.418)		(0.406)
Harvey, storm fixed effect	0.201	0.264	0.195	0.568	0.198
	(0.363)	(0.399)	(0.364)	(0.473)	(0.365)
Irma, storm fixed effect	0.296	0.281	0.243	0.160	0.317
	(0.253)	(0.276)	(0.251)	(0.233)	(0.254)
Isaias, storm fixed effect	−0.551*	−0.480*	−0.564*	−0.504*	−0.513*
	(0.174)	(0.200)	(0.186)	(0.187)	(0.174)
Laura, storm fixed effect	−0.120	−0.306	−0.179	0.077	−0.069
	(0.353)	(0.392)	(0.360)	(0.394)	(0.354)
Michael, storm fixed effect	0.042	0.040	−0.016	−0.141	0.079
	(0.265)	(0.291)	(0.263)	(0.245)	(0.266)
Sally, storm fixed effect	0.209	0.111	0.130	0.168	0.255
	(0.284)	(0.313)	(0.283)	(0.277)	(0.285)
Zeta, storm fixed effect	−0.145	−0.365	−0.185	−0.124	−0.121
	(0.267)	(0.295)	(0.267)	(0.248)	(0.268)
Wind speed 34 knots, fixed effect	0.071	0.030	0.137		0.102
	(0.145)	(0.177)	(0.164)		(0.144)
Wind speed 50 knots, fixed effect	0.525*	0.354	0.595*	0.539*	0.556*
	(0.191)	(0.248)	(0.215)	(0.102)	(0.190)
Wind speed 64 knots, fixed effect	0.710*	0.366	0.749*	0.742*	0.756*
	(0.253)	(0.345)	(0.284)	(0.172)	(0.252)
log(peak outages), spatial lag	0.411*	0.358*	0.418*	0.341*	0.406*
	(0.057)	(0.062)	(0.058)	(0.071)	(0.057)
log(population), spatial lag	−0.282*	−0.151	−0.247*	−0.174*	−0.245*
	(0.077)	(0.091)	(0.081)	(0.087)	(0.072)
log(county size), spatial lag	−0.306*	−0.325*	−0.175	−0.372*	−0.289*
	(0.130)	(0.163)	(0.139)	(0.140)	(0.129)
Ratio of customer served by IOU, spatial lag	0.182	0.119	0.068	0.355	0.222
	(0.159)	(0.196)	(0.170)	(0.183)	(0.159)

FSF flood risk score, spatial lag	−0.116*	−0.138*	−0.090	−0.098	−0.122*
	(0.058)	(0.069)	(0.063)	(0.063)	(0.058)
Socioeconomic Vulnerability, spatial lag	−0.441	0.587	0.229	0.081	−0.079
	(0.414)	(0.522)	(0.453)	(0.468)	(0.301)
Household Characteristics Vulnerability, spatial lag	−0.193	−0.500	−0.371	−0.258	
	(0.224)	(0.295)	(0.243)	(0.252)	
Racial & Ethnic Minority Vulnerability, spatial lag	−0.061	−0.110	0.095	0.212	
	(0.263)	(0.334)	(0.286)	(0.312)	
Housing Type & Transportation Vulnerability, spatial lag	0.582*	−0.085	0.122	0.192	
	(0.238)	(0.295)	(0.252)	(0.245)	
Arkansas, state spatial lag	0.811	1.830	0.402	0.880	0.934
	(1.241)	(1.373)	(1.343)	(1.368)	(1.245)
Florida, state spatial lag	−0.523	−0.676	−0.371	−0.247	−0.333
	(0.351)	(0.477)	(0.389)	(0.367)	(0.347)
Georgia, state spatial lag	0.231	0.289	0.325	0.520	0.342
	(0.307)	(0.421)	(0.344)	(0.328)	(0.303)
Louisiana, state spatial lag	−0.003	1.550	−0.101	0.332	0.130
	(1.079)	(1.095)	(1.170)	(1.189)	(1.077)
Mississippi, state spatial lag	−0.175	−0.255	0.010	0.104	−0.064
	(0.782)	(0.813)	(0.756)	(0.701)	(0.782)
North Carolina, state spatial lag	1.364	0.989	1.375	1.281	1.396
	(0.837)	(1.054)	(0.925)	(0.771)	(0.839)
South Carolina, state spatial lag	1.081*	1.176	1.035	1.368*	1.138*
	(0.542)	(0.755)	(0.601)	(0.513)	(0.544)
Wind speed 34 knots, spatial lag	0.070	0.411*	0.072		0.021
	(0.174)	(0.208)	(0.192)		(0.171)
Wind speed 50 knots, spatial lag	0.144	0.673*	0.066	−0.207	0.059
	(0.242)	(0.315)	(0.270)	(0.151)	(0.239)
Wind speed 64 knots, spatial lag	0.329	1.077*	0.247	−0.069	0.219
	(0.331)	(0.444)	(0.368)	(0.249)	(0.328)
Constant	4.971*	4.162*	3.505*	4.155*	4.602*
	(1.160)	(1.381)	(1.200)	(1.196)	(1.109)
ρ	0.381*	0.400*	0.406*	0.488*	0.385*
	(0.036)	(0.032)	(0.034)	(0.039)	(0.036)
Observations	862	1,104	943	691	862
Log Likelihood	-975.870	-1,633.431	-1,173.041	-718.029	-981.319
σ^2	0.541	1.080	0.673	0.441	0.547
Akaike Inf. Crit.	2,049.739	3,364.862	2,444.081	1,528.058	2,048.638
Wald Test (df = 1)	112.260*	156.148*	143.638*	155.456*	114.859*
LR Test (df = 1)	103.907*	138.688*	126.881*	126.783*	105.700*

Note: * $p<0.05$,
ρ: spatial lag term of dependent variable

Table S2. Spatial Durbin model with individual hurricanes

	Dependent variable:
	log(outage duration)
log(peak outages)	0.358*
	(0.035)
log(population)	−0.335*
	(0.048)
log(county size)	0.162*
	(0.076)
Ratio of customer served by IOU	0.083
	(0.101)
FSF flood risk score	0.030
	(0.037)
Socioeconomic Vulnerability of Florence	0.498
	(0.533)
Socioeconomic Vulnerability of Harvey	1.147
	(0.964)
Socioeconomic Vulnerability of Irma	0.342
	(0.274)
Socioeconomic Vulnerability of Isaias	−0.672
	(0.643)
Socioeconomic Vulnerability of Laura	1.026†
	(0.572)
Socioeconomic Vulnerability of Michael	1.394*
	(0.378)
Socioeconomic Vulnerability of Sally	1.334*
	(0.564)
Socioeconomic Vulnerability of Zeta	0.439
	(0.361)
Household Characteristics Vulnerability	−0.182
	(0.119)
Racial & Ethnic Minority Vulnerability	−0.147
	(0.168)
Housing Type & Transportation Vulnerability	−0.055
	(0.130)
Arkansas, state fixed effect	−0.684
	(1.177)
Florida, state fixed effect	0.187
	(0.319)
Georgia, state fixed effect	−0.233
	(0.264)
Louisiana, state fixed effect	0.127
	(1.032)
Mississippi, state fixed effect	0.068
	(0.729)
North Carolina, state fixed effect	−1.216
	(0.747)
South Carolina, state fixed effect	−1.189*
	(0.499)
Tennessee state, fixed effect	1.515*
	(0.541)
Harvey, storm fixed effect	−1.877
	(1.506)
Irma, storm fixed effect	1.561*
	(0.648)
Isaias, storm fixed effect	0.450
	(1.013)
Laura, storm fixed effect	1.545
	(1.107)
Michael, storm fixed effect	0.726

	(0.749)
Sally, storm fixed effect	1.428
	(0.960)
Zeta, storm fixed effect	0.952
	(0.695)
Wind speed 34 knots, fixed effect	0.038
	(0.144)
Wind speed 50 knots, fixed effect	0.514*
	(0.189)
Wind speed 64 knots, fixed effect	0.736*
	(0.250)
log(peak outages), spatial lag	0.390*
	(0.058)
log(population), spatial lag	−0.284*
	(0.079)
log(county size), spatial lag	−0.283*
	(0.132)
Ratio of customer served by IOU, spatial lag	0.213
	(0.158)
FSF flood risk score, spatial lag	−0.145*
	(0.059)
Socioeconomic Vulnerability of Florence, spatial lag	0.811
	(0.904)
Socioeconomic Vulnerability of Harvey, spatial lag	2.684*
	(1.364)
Socioeconomic Vulnerability of Irma, spatial lag	−0.683
	(0.537)
Socioeconomic Vulnerability of Isaias, spatial lag	0.529
	(1.345)
Socioeconomic Vulnerability of Laura, spatial lag	−1.820
	(1.132)
Socioeconomic Vulnerability of Michael, spatial lag	−0.978
	(0.630)
Socioeconomic Vulnerability of Sally, spatial lag	−1.589
	(1.064)
Socioeconomic Vulnerability of Zeta, spatial lag	−0.542
	(0.648)
Household Characteristics Vulnerability, spatial lag	−0.201
	(0.226)
Racial & Ethnic Minority Vulnerability, spatial lag	0.016
	(0.266)
Housing Type & Transportation Vulnerability, spatial lag	0.587*
	(0.237)
Arkansas, state spatial lag	0.756
	(1.226)
Florida, state spatial lag	−0.686
	(0.355)
Georgia, state spatial lag	0.232
	(0.306)
Louisiana, state spatial lag	0.006
	(1.063)
Mississippi, state spatial lag	−0.155
	(0.772)
North Carolina, state spatial lag	1.323
	(0.826)
South Carolina, state spatial lag	0.994
	(0.537)
Wind speed 34 knots, spatial lag	0.065
	(0.174)
Wind speed 50 knots, spatial lag	0.148
	(0.241)

Wind speed 64 knots, spatial lag	0.259
	(0.329)
Constant	4.079*
	(1.275)
ρ	0.385*
	(0.036)
Observations	862
Log Likelihood	-963.188
σ^2	0.525
Akaike Inf. Crit.	2,052.377
Wald Test	115.436* (df = 1)
LR Test	104.919* (df = 1)

Note: †p< 0.1, *p<0.05

Table S3. Spatial Durbin model with gulf hurricanes

	Dependent variable:
	log(outage duration)
log(peak outages)	0.349^{*}
	(0.035)
log(population)	-0.325^{*}
	(0.048)
log(county size)	0.142
	(0.077)
Ratio of customer served by IOU	0.078
	(0.102)
FSF flood risk score	0.020
	(0.037)
Socioeconomic Vulnerability of gulf hurricanes	0.650^{*}
	(0.221)
Socioeconomic Vulnerability of non-gulf hurricanes	0.021
	(0.439)
Household Characteristics Vulnerability	−0.199
	(0.120)
Racial & Ethnic Minority Vulnerability	−0.113
	(0.169)
Housing Type & Transportation Vulnerability	−0.041
	(0.131)
Arkansas, state fixed effect	−0.663
	(1.190)
Florida, state fixed effect	0.055
	(0.315)
Georgia, state fixed effect	−0.262
	(0.265)
Louisiana, state fixed effect	0.132
	(1.044)
Mississippi, state fixed effect	0.065
	(0.738)
North Carolina, state fixed effect	−1.345
	(0.754)
South Carolina, state fixed effect	-1.277^{*}
	(0.503)
Tennessee state, fixed effect	0.653
	(0.406)
Harvey, storm fixed effect	0.960
	(0.619)
Irma, storm fixed effect	1.078
	(0.568)
Isaias, storm fixed effect	-0.569^{*}
	(0.174)
Laura, storm fixed effect	0.655
	(0.619)
Michael, storm fixed effect	0.830
	(0.575)
Sally, storm fixed effect	0.993
	(0.580)
Zeta, storm fixed effect	0.635
	(0.573)
Wind speed 34 knots, fixed effect	0.072
	(0.145)
Wind speed 50 knots, fixed effect	0.529^{*}
	(0.191)
Wind speed 64 knots, fixed effect	0.711^{*}
	(0.252)
log(peak outages), spatial lag	0.413^{*}

	(0.057)
log(population), spatial lag	-0.284^{*}
	(0.078)
log(county size), spatial lag	-0.270^{*}
	(0.132)
Ratio of customer served by IOU, spatial lag	0.197
	(0.159)
FSF flood risk score, spatial lag	-0.130^{*}
	(0.059)
Socioeconomic Vulnerability of gulf hurricanes, spatial lag	−0.615
	(0.424)
Socioeconomic Vulnerability of non-gulf hurricanes, spatial lag	1.017
	(0.778)
Household Characteristics Vulnerability, spatial lag	−0.214
	(0.224)
Racial & Ethnic Minority Vulnerability, spatial lag	−0.052
	(0.263)
Housing Type & Transportation Vulnerability, spatial lag	0.551^{*}
	(0.238)
Arkansas, state spatial lag	0.811
	(1.238)
Florida, state spatial lag	−0.536
	(0.350)
Georgia, state spatial lag	0.251
	(0.306)
Louisiana, state spatial lag	0.001
	(1.075)
Mississippi, state spatial lag	−0.155
	(0.780)
North Carolina, state spatial lag	1.423
	(0.835)
South Carolina, state spatial lag	1.097^{*}
	(0.541)
Wind speed 34 knots, spatial lag	0.048
	(0.173)
Wind speed 50 knots, spatial lag	0.114
	(0.242)
Wind speed 64 knots, spatial lag	0.291
	(0.331)
Constant	4.176^{*}
	(1.236)
ρ	0.385^{*}
	(0.036)
Observations	862
Log Likelihood	-973.380
σ^2	0.537
Akaike Inf. Crit.	2,048.760
Wald Test	115.063^{*} (df = 1)
LR Test	105.881^{*} (df = 1)
Note:	$^{*}p<0.05$

References

1. L Anselin, *Spatial econometrics: methods and models.* (Springer Science & Business Media) Vol. 4, (1988).
2. L Anselin, AK Bera, R Florax, MJ Yoon, Simple diagnostic tests for spatial dependence. *Reg. science urban economics* **26**, 77–104 (1996).
3. RS Bivand, E Pebesma, V Gómez-Rubio, *Applied spatial data analysis with R, Second edition.* (Springer, NY), (2013).
4. JP LeSage, An introduction to spatial econometrics. *Revue d'économie industrielle* pp. 19–44 (2008).
